# Association between estradiol levels in early pregnancy and risk of preeclampsia after frozen embryo transfer

**DOI:** 10.3389/fendo.2023.1223181

**Published:** 2023-09-15

**Authors:** Yun-Chiao Hsieh, Tzu-Ching Kao, Ih-Jane Yang, Po-Kai Yang, Kuang-Han Chao, Mei-Jou Chen, Jehn-Hsiahn Yang, Shee-Uan Chen

**Affiliations:** ^1^ Department of Obstetrics and Gynecology, National Taiwan University Hospital, Hsinchu, Taiwan; ^2^ Department of Obstetrics and Gynecology, National Taiwan University Hospital, Taipei, Taiwan; ^3^ Department of Obstetrics and Gynecology, National Taiwan University Hospital, Yunlin, Taiwan; ^4^ Livia Shangyu Wan Chair Professor of Obstetrics and Gynecology, College of Medicine, National Taiwan University, Taipei, Taiwan

**Keywords:** preeclampsia, estradiol, early pregnancy, frozen embryo transfer, programmed cycle

## Abstract

**Introduction:**

The failure of remodeling the spiral arteries is associated with the pathogenesis of preeclampsia. Estradiol (E2) plays a crucial role in placentation and may be involved in the development of preeclampsia. However, there is a lack of data in this area. This study aims to assess the association between serum estradiol levels in early pregnancy and the risk of preeclampsia.

**Methods:**

We conducted a retrospective cohort study on patients who conceived after frozen embryo transfer (FET) using data from a database at a university-affiliated *in vitro* fertilization center. The study period spanned from January 1, 2010, to December 31, 2020. Multivariable logistic regression analyses were performed to determine the adjusted effect of E2 levels on the risk of preeclampsia. We compared the odds ratios of preeclampsia across quartiles of E2 levels and assessed their significance.

**Results:**

Serum E2 levels at the fifth gestational week were significantly different between women with and without preeclampsia after FET programmed cycles (607.5 ± 245.4 vs. 545.6 ± 294.4 pg/ml, p=0.009). A multivariable logistic regression model demonstrated that E2 levels in early pregnancy were independent risk factors for preeclampsia. We observed an increased odds ratio of preeclampsia with increasing quartiles of estradiol levels after adjusting for potential confounders in FET programmed cycles. When comparing quartiles 3 and 4 (E2 > 493 pg/ml at the fifth gestational week) to quartiles 1 and 2, the odds ratios of preeclampsia were significantly higher.

**Conclusion:**

We found that serum E2 levels in early pregnancy may impact the risk of preeclampsia, particularly following FET programmed cycles. The association between E2 levels in early pregnancy and preeclampsia deserves further investigation.

## Introduction

Preeclampsia is a major cause of maternal mortality and morbidities, perinatal deaths, preterm birth, and intrauterine growth restriction which complicates 2 – 8% of pregnancies globally (1).

The pathophysiology of preeclampsia involves both maternal and fetal/placental factors. Disturbances in the development of placental vasculature early in pregnancy may lead to relative placental hypo-perfusion, which then results in release of antiangiogenic factors into the maternal circulation that affect the maternal systemic endothelial function and cause hypertension and other manifestations of the disease. However, the trigger for abnormal placental development and the subsequent cascade of events remains unknown.

Estradiol (E2) is a key regulator of trophoblast invasion and remodeling of uterine decidual spiral arterioles during placentation, but there is little research on the effects of E2 in early pregnancy on placentation and subsequent preeclampsia (2–4). Excessive E2 inhibited the proliferation of decidualized endometrial stromal cells in rodent models, resulting in less development of the spiral arteries, which was supposed to be one of the important factors of developing preeclampsia (5). Several pathophysiologic mechanisms of preeclampsia, including the imbalance of angiogenic factors, nitric oxide synthesis deficiency, and impaired maternal vascular endothelial functions, might also be modulated by E2 (6–8).

The use of frozen embryo transfer (FET) has increased dramatically worldwide with the improvements in vitrification technology over the past decade. In the United States, the percentage of the FET cycle has increased from 26.3% in 2011 to 51.7% in 2018 (9). Therefore, investigating the potential effect of E2 levels on the preeclampsia risk will provide information for predicting preeclampsia or improving obstetric outcomes when prescribing E2 replacement for luteal support or early pregnancy support in FET cycles. The goal of this study was to assess the association between E2 levels in early pregnancy and the risk of preeclampsia following FET.

## Materials and methods

### Study population

The data were obtained from electronic medical records at the National Taiwan University Hospital (NTUH). The study included patients who conceived after FET and had one gestational sac at 5 weeks of pregnancy and singleton deliveries between January 1, 2010, and December 31, 2020. To ensure accuracy, the data on FET cycle regimens and obstetric complications were validated by closely monitoring all patients’ prenatal examinations at NTUH’s outpatient department. Patients with pre-existing chronic hypertension (HTN), diabetes mellitus (DM), previous preeclampsia, and gestational diabetes mellitus (GDM) were excluded from the study.

### Regimens of FET cycles

In FET programmed cycles, oral E2 (ESTRADE®, estradiol valerate, 2mg/tablet, Synmosa, Taipei, Taiwan) was exclusively used for endometrial preparation. The dosage of estradiol valerate ranged from 2 tablets to 3 tablets twice a day, depending on the endometrial thickness and physician’s experience. The treatment was continued until the 8th or 9th gestational week. Oral, injectable, and transvaginal progesterone were administered for endometrial maturation and luteal phase support.

In FET natural cycles, patients did not receive any medications prior to the endogenous luteinizing hormone surge and did not require any medications for luteal support. The serum E2 levels observed in these cycles were entirely endogenous, reflecting the levels seen in normal conceptions during early pregnancy.

### Definition of preeclampsia

Preeclampsia was defined as having blood pressure levels of 140 mm Hg systolic or 90 mm Hg diastolic or higher on two or more occasions, spaced more than 6 hours apart, accompanied by proteinuria or end organ dysfunction. Alternatively, it was defined as having blood pressure levels of 160 mm Hg systolic or 110 mm Hg diastolic or higher, following the standard criteria provided by the American College of Obstetrics and Gynecology at the time of the study. All diagnoses were made by medical doctors.

### E2 measurements

Serum samples were analyzed using the Immulite 2000 reproductive hormone assays (Diagnostic Product Corporation, Siemens, Los Angeles, CA, USA), a random-access immunoassay system utilizing enzyme-amplified chemiluminescence immunoassay (CLIA) technology. The sensitivity of the assay for E2 was 15 pg/mL. The intra-assay and inter-assay coefficients of variation were 6.7% and 9.7%, respectively. Serum E2 levels for all women who conceived through assisted reproductive technology at our institution were routinely measured on two specific days: ‘OV + 16’ (approximately the fourth gestational week) and ‘OV + 22’ (approximately the fifth gestational week).

In this study, ‘OV’ referred to the day when progesterone administration commenced in FET programmed cycles or the day when progesterone was measured to be around 1.0 ng/mL with the dominant follicle disappearing in FET natural cycles.

### Endometrial thickness measurements

Endometrial thickness was measured prior to the initiation of progesterone in programmed FET cycles using transvaginal ultrasound (TOSHIBA Nemio XG [SSA-580A], Tokyo, Japan; probe PVM-651VT, 6MHz). To obtain an accurate measurement, a transvaginal ultrasound probe was placed perpendicular to the uterine midline, and measurements were taken in the sagittal plane. The focus was on the thickest area of the endometrium, with calipers placed at opposite points of the anterior and posterior endometrial-myometrial interfaces. The total double-layer thickness in millimeters was calculated.

### Statistical analyses

Descriptive statistics were reported as number (n) and percentages for categorical variables, while mean and standard deviation (SD) were used for continuous variables. Differences between groups were assessed using the chi-squared test for dichotomous variables, and the T-test and Mann–Whitney test for continuous variables.

To account for potential confounding factors between E2 levels and the odds ratio of preeclampsia, multivariable logistic regression analysis was performed. As the exposure–disease relationship for hormones may not be linear or monotonic, E2 levels were categorized into quartiles at the fifth gestational weeks.

All statistical analyses were conducted using the SPSS software program, version 22.0 (SPSS, Chicago, IL, USA). A significance level of P < 0.05 was considered statistically significant.

## Results

A total of 888 Taiwanese women who met the inclusion criteria were included in the study, of whom 95 were diagnosed with preeclampsia. **Table 1** presents the background characteristics of women with and without preeclampsia. There were no significant differences in age, proportion of maternal polycystic ovarian syndrome (PCOS), aspirin usage during pregnancy, causes of infertility, progesterone levels in early pregnancy, and endometrial thickness. However, women with preeclampsia had a higher mean body mass index (BMI) compared to those without preeclampsia (26.6 ± 4.9 vs. 24.7 ± 3.7, p=0.001). Additionally, nulliparity was more common in women with preeclampsia compared to controls (83.2% vs. 67.3%, p=0.002). Furthermore, FET programmed cycles were more prevalent in women with preeclampsia compared to those without preeclampsia (75.8% vs. 65.1%, p=0.037). Serum E2 levels at the fourth (462.1 ± 250.6 vs. 393.7 ± 234.3 pg/mL, p=0.007) and fifth gestational weeks (570.3 ± 246.3 vs. 510.0 ± 280.5 pg/mL, p=0.005) were significantly different between women with and without preeclampsia.

**Table 1:** Maternal and treatment characteristics of women with or without preeclampsia.

	Without preeclampsia (N=793)	With preeclampsia (N=95)	P-value
**Maternal age, years**	36.0 ± 4.1	36.5 ± 5.1	0.27
**Maternal BMI (kg/m2)^#^**	24.7 ± 3.7	26.6 ± 4.9	0.001
**Smoking**	0	0	NA
**Alcohol drinking**	0	0	NA
**Aspirin during pregnancy**	75 (9.5%)	13 (13.7%)	0.193
**PCOS**	110 (13.9%)	15 (15.8%)	0.611
**Parity**			0.002
Nulliparity	534 (67.3%)	79 (83.2%)	
Multiparity	259 (32.7%)	16 (16.8%)	
**Gestational age at birth**	37.6 ± 2.4	36.6 ± 1.7	0.001
**Embryo stage at transfer**			0.195
Cleavage	259 (32.7%)	30 (31.6%)	
Blastocyst	534 (67.3%)	65 (68.4%)	
**Type of cryopreservation**			0.966
Slow freezing	93 (11.7%)	11 (11.6%)	
Vitrification	700 (88.3%)	84 (88.4%)	
**Cause of infertility**			
Anovulation	135 (17.0%)	18 (18.9%)	0.643
Tubal factor	148 (18.7%)	20 (21.1%)	0.578
Male factor	246 (31.1%)	29 (30.5%)	0.915
Uterine factor	37 (4.7%)	5 (5.3%)	0.798
Endometriosis	50 (6.3%)	6 (6.3%)	0.222
DOR	166 (21.0%)	28 (29.5%)	0.058
Unexplained	87 (11.0%)	6 (6.3%)	0.160
Others*	64 (8.1%)	11 (11.6%)	0.247
**Fertilization methods**			0.858
IVF	113 (14.2%)	14 (14.7%)	
ICSI	522 (65.8%)	60 (63.2%)	
Both IVF & ICSI	158 (19.9%)	21 (22.1%)	
**Regimens of FET cycles**			0.037
Natural cycle	277 (34.9%)	23 (24.2%)	
Programmed cycle	516 (65.1%)	72 (75.8%)	
**Estradiol level (pg/ml)**			
E2 levels at 3^th^ GW	346.0 ± 255.4	378.8 ± 213.7	0.230
E2 levels at 4^th^ GW	393.7 ± 234.3	462.1 ± 250.6	0.007
E2 levels at 5^th^ GW	510.0 ± 280.5	570.3 ± 246.3	0.005
**Progesterone level (ng/mL)**			
P4 levels at 3^th^ GW	18.7 ± 21.2	16.7 ± 22.0	0.382
P4 levels at 4^th^ GW	23.3 ± 28.0	19.2 ± 19.4	0.160
P4 levels at 5^th^ GW	26.1 ± 36.4	22.9 ± 35.0	0.416
Endometrial thickness (mm)	10.7 ± 1.98	10.7 ± 1.93	0.865

BMI, body mass index; DOR, diminished ovarian reserve; E2, estradiol; FET, frozen embryo transfer; GW, gestational week; ICSI, intracytoplasmic sperm injection; IVF, *in vitro* fertilization; NA, not applicable; P4, progesterone; PCOS, polycystic ovary syndrome.

# Pre-pregnancy BMI.

* The ‘others’ category of causes of infertility included social or medical oocyte/embryo freezing, preimplantation genetic testing for monogenic diseases, chromosomal structural rearrangements, and aneuploidies.

**Table 2** presents the results of multivariable logistic regression analyses, identifying risk factors for preeclampsia in FET. After adjusting for maternal BMI, nulliparity, FET regimens, and E2 levels at the fourth and fifth gestational weeks, higher maternal BMI (adjusted odds ratio [aOR] 1.13, 95% confidence interval [CI] 1.07-1.20), nulliparity (aOR 2.54, 95% CI 1.44-4.48), and higher E2 levels at the fourth and fifth gestational weeks (aOR 1.08, 95% CI 1.01-1.16) were associated with an increased odds ratio of preeclampsia. FET regimens did not show independent association with the odds of preeclampsia after controlling for other confounders.

**Table 2:** Multivariable logistic regression analyses of the risk factors for preeclampsia in FET.

Risk factors	Crude OR (95%CI)	Adjusted OR^*^(95%CI)	P-value
Maternal BMI (kg/m2)	1.13 (1.07-1.19)	1.13 (1.07-1.20)	0.001
Nulliparity	2.40 (1.37-4.18)	2.54 (1.44-4.48)	0.001
Programmed cycle	1.68 (1.03-2.75)	1.38 (0.81-2.34)	0.232
E2 levels at 4th GW	1.10 (1.02-1.84)		
E2 levels at 5th GW	1.07 (1.01-1.14)		
E2 levels at 4-5th GW†	1.08 (1.02-1.14)	1.08 (1.01-1.16)	0.025

*Adjust for BMI, nulliparity, the regimen of FET cycles and E2 levels at 4-5th GW.

**†**Generalized estimating equations models have been used as they allow adjustment of within-individual correlation.

BMI, body mass index; CI, confidence interval; E2, estradiol; FET, frozen embryo transfer; GW, gestational week; OR, odds ratio.

When stratified by FET cycles, **Table 3** compares E2 levels at the fourth and fifth gestational weeks between women with and without preeclampsia. Significantly higher E2 levels at the fifth gestational weeks were observed in women with preeclampsia compared to those without preeclampsia in FET programmed cycles (607.5 ± 245.4 vs. 545.6 ± 294.4, p=0.009) but not in the FET natural cycles.

**Table 3:** Comparison of estradiol levels (pg/ml) in different FET cycles.

	Total (N=888)	Without preeclampsia (N=793)	With preeclampsia (N=95)	P-value
**Natural cycle (n)**	300	277	23	
E2 levels at 4^th^ GW	283.4 ± 149.9	280.1 ± 145.7	323.1 ± 192.8	0.186
E2 levels at 5^th^ GW	450.3 ± 244.1	449.5 ± 246.4	459.7 ± 218.5	0.848
**Programmed cycle (n)**	588	516	72	
E2 levels at 4^th^ GW	462.0 ± 251.3	456.8 ± 252.4	499.6 ± 241.9	0.087
E2 levels at 5^th^ GW	553.2 ± 289.4	545.6 ± 294.4	607.5 ± 245.4	0.009

E2, estradiol; FET, frozen embryo transfer; GW, gestational week.

**Table 4** displays the odds ratios of preeclampsia stratified by E2 levels quartiles in FET programmed cycles at the fifth gestational weeks. A statistically significant increased odds ratio of preeclampsia was observed in quartiles 3 and 4 (E2 > 493 pg/mL) compared to quartiles 1 and 2 (aOR 2.13-2.42, p<0.05).

**Table 4:** Odds ratio of preeclampsia by estradiol levels quartiles in FET programmed cycles (N=588).

5^th^ GW E2 levels quartiles, (pg/ml)	n	Women with preeclampsia (n=72) n (%, per group)	*aOR (95%CI)	*aOR (95%CI)	*aOR (95%CI)
≤ 350	147	12 (8.2%)	1.0		
351 – 493	147	13 (8.8%)	1.04 (0.45 – 2.42)	1.0	
494 – 678	147	24 (16.3%)	2.15 (1.02 – 4.56)	2.13 (1.01 – 4.46)	1.0
> 678	147	23 (15.6%)	2.36 (1.10 – 5.05)	2.42 (1.13 – 5.15)	1.12 (0.59 – 2.13)

*Adjust for BMI, nulliparity

aOR, adjusted odds ratio; BMI, body mass index; CI, confidence interval; E2, estradiol; GW, gestational week; FET, frozen embryo transfer; OR, odds ratio; PCOS, polycystic ovary syndrome.

## Discussion

It was observed in this retrospective cohort study that increasing E2 levels in early pregnancy were associated with an increased odds ratio of preeclampsia after FET. These findings support the concept that E2 levels may have an effect on placentation and subsequent obstetric outcomes in animal models (5).

The role of E2 in regulating placentation has been the subject of controversy. According to an *in vitro* trophoblast model, human chorionic gonadotropin and progesterone, but not E2, play direct roles in controlling trophoblast movement during vascular remodeling in early pregnancy (9). However, E2 may affect angiogenic markers such as vascular endothelial growth factor, placental growth factor, and endothelial nitric oxide synthase, which are involved in angiogenic processes (8, 10, 11). It has been hypothesized that low levels of E2 may lead to insufficient trophoblast development and angiogenesis (4). Conversely, prematurely increasing E2 in early pregnancy has been shown to suppress extra-villous cytotrophoblast invasion and remodeling of the uterine spiral arteries in animal models, as indicated by other studies (12–14).

According to a small case-control study, E2 levels in early pregnancy (median 9.8 weeks, range 5.3–15.3 weeks) were found to be higher in preeclamptic pregnancies compared to control pregnancies after fresh embryo transfer (3). Additionally, several retrospective studies have reported that elevated peak serum E2 levels during controlled ovarian hyperstimulation were associated with a higher risk of preeclampsia and abnormal placental implantation after fresh embryo transfer (15, 16). These findings align with a previous animal study that revealed higher E2 levels have a negative impact on the arterial modeling of the uterus during early pregnancy (17).

Our results are consistent with several recent retrospective studies indicating that the risk of preeclampsia is higher in FET programmed cycles compared to FET natural cycles (18–22). We also observed that E2 levels in early pregnancy were significantly higher in FET programmed cycles compared to FET natural cycles. However, after adjusting for E2 levels, FET programmed cycles were not independently associated with the odds of preeclampsia. This suggests that the E2 levels may be the true factor influencing the odds ratio of preeclampsia after FET, regardless of the choice of protocol.

Taken together, E2 is critical to placentation. It has been hypothesized that low but adequate levels of E2 exhibited during early pregnancy are required to allow the normal progression of trophoblast vascular invasion. Additionally, it has been suggested that the elevation of E2 levels in the late first trimester plays a physiological role in suppressing further arterial trophoblast invasion (14). Our findings support a part of this hypothesis: excessive E2 levels in early pregnancy may have negative effects on placentation.

Further larger studies with comprehensive steroid profiling are required to investigate the potential association between supraphysiological E2 levels in FET and abnormal placentation. It is also crucial to determine whether this association independently contributes to the development of preeclampsia, irrespective of other influencing factors. These studies would provide valuable insights into the role of E2 in placental function and its impact on pregnancy outcomes, helping to improve our understanding of the underlying mechanisms and guide clinical management strategies.

This study had several strengths. Firstly, all cases were followed at a single institution, ensuring consistent data collection and management. Additionally, the medications prescribed during FET cycles and pregnancies were well-documented, allowing for adjustments of potential confounding factors such as aspirin use during pregnancy, prior history of preeclampsia or gestational diabetes mellitus (GDM), and history of polycystic ovary syndrome (PCOS), diabetes mellitus (DM), or hypertension (HTN). In the case of FET programmed cycles, the serum E2 levels were solely attributed to prescribed E2 supplementation, with estradiol valerate specifically used for endometrial preparation and early gestational weeks. This approach reduces the risk of bias when evaluating serum E2 levels.

However, this study also had some limitations. Firstly, its retrospective design inherently carried biases and limitations that may have affected the findings. Not all confounding factors were adjusted for, and there is a possibility of residual confounding even after conducting multivariate logistic regression analysis. Although the results were statistically significant, caution should be exercised in their interpretation due to the complexity of preeclampsia as a multifactorial outcome that cannot be solely explained by a single factor. The effects of E2 on placentation are likely to be complicated and influenced by the specific levels and timing of E2 exposure.

It is worth noting that while some studies have indicated a potential contribution of progesterone to placentation (2, 9, 23, 24), this study did not observe an effect of progesterone on the risk of preeclampsia after FET.

## Data availability statement

The raw data supporting the conclusions of this article will be made available by the authors, without undue reservation.

## Ethics statement

The studies involving humans were approved by The study was approved by the ethics committees at the National Taiwan University Hospital (202109103RIND). The studies were conducted in accordance with the local legislation and institutional requirements. Written informed consent for participation was not required from the participants or the participants’ legal guardians/next of kin in accordance with the national legislation and institutional requirements.

## Author contributions

YC-H. performed the statistical analysis and wrote the manuscript; T-CK, I-JY, P-KY, K-HC, M-JC, J-HY, and S-UC reviewed and edited the manuscript. M-JC and S-UC designed research. All authors contributed to the article and approved the submitted version.
